# Non-invasive Presymptomatic Detection of *Cercospora beticola* Infection and Identification of Early Metabolic Responses in Sugar Beet

**DOI:** 10.3389/fpls.2016.01377

**Published:** 2016-09-22

**Authors:** Nadja Arens, Andreas Backhaus, Stefanie Döll, Sandra Fischer, Udo Seiffert, Hans-Peter Mock

**Affiliations:** ^1^Applied Biochemistry, Department of Physiology and Cell Biology, Leibniz Institute of Plant Genetics and Crop Plant ResearchGatersleben, Germany; ^2^Biosystems Engineering, Fraunhofer Institute for Factory Operation and AutomationMagdeburg, Germany; ^3^Strube Research GmbH & Co. KGSöllingen, Germany

**Keywords:** phenotyping, hyperspectral imaging, metabolic profiling, metabolomics, *Beta vulgaris*, LC–MS

## Abstract

*Cercospora beticola* is an economically significant fungal pathogen of sugar beet, and is the causative pathogen of *Cercospora* leaf spot. Selected host genotypes with contrasting degree of susceptibility to the disease have been exploited to characterize the patterns of metabolite responses to fungal infection, and to devise a pre-symptomatic, non-invasive method of detecting the presence of the pathogen. Sugar beet genotypes were analyzed for metabolite profiles and hyperspectral signatures. Correlation of data matrices from both approaches facilitated identification of candidates for metabolic markers. Hyperspectral imaging was highly predictive with a classification accuracy of 98.5–99.9% in detecting *C. beticola*. Metabolite analysis revealed metabolites altered by the host as part of a successful defense response: these were L-DOPA, 12-hydroxyjasmonic acid 12-*O*-β-D-glucoside, pantothenic acid, and 5-*O*-feruloylquinic acid. The accumulation of glucosylvitexin in the resistant cultivar suggests it acts as a constitutively produced protectant. The study establishes a proof-of-concept for an unbiased, presymptomatic and non-invasive detection system for the presence of *C. beticola*. The test needs to be validated with a larger set of genotypes, to be scalable to the level of a crop improvement program, aiming to speed up the selection for resistant cultivars of sugar beet. Untargeted metabolic profiling is a valuable tool to identify metabolites which correlate with hyperspectral data.

## Introduction

The fungal pathogen *Cercospora beticola*, the causative organism of the sugar beet leaf disease *Cercospora* leaf spot, imposes a major constraint over the crop’s yield worldwide. The pathogen employs the photosensitizer cercosporin to induce necrotic lesions on the leaves which impair the photosynthetic performance and therefore negatively affect taproot yield (Staerkel et al., 2013). Host variations of resistance are known, but its physiological basis is not fully understood. The genetic basis of this resistance is very often complex, although there is one example known of a race-specific, monogenic resistance (Lewellen and Whitney, 1976; Whitney and Lewellen, 1976; Koch et al., 2000). Based on the observation that effectors of *C. beticola* suppress the transcription of the host’s gene encoding phenylalanine ammonia lyase, it has been proposed that phenylpropanoids are involved in the defense response (Schmidt et al., 2004, 2008). Thus adopting a metabolomic approach to characterize the resistance reaction may well-prove to be highly informative.

Two generalized strategies are followed in metabolomic profiling: the first is non-targeted in the sense that an attempt is made to identify as many metabolites as possible, thereby enabling the comparison of metabolite profiles and identification of disease resistance marker; in contrast the targeted approach focuses on quantifying a set of pre-determined compounds. The physiological status of the leaf in the course of a compatible (susceptible) and incompatible (resistant) host–pathogen interaction has been successfully monitored in some detail using the former approach (Fiehn, 2002; Viant and Sommer, 2013). Metabolomic profiling using reverse phase liquid chromatography coupled with high resolution mass spectrometry is commonly applied for the detection of semi-polar compounds. Complex matrices (e.g., plant extracts) are separated by polarity and mass/charge (*m/z*) values are determined. Software for automated peak detection, deconvolution and alignment is used to set up a data matrix for multivariate statistic analysis.

Traditionally, strategies to minimize yield loss comprise either in breeding efforts for resistant cultivars or the application of fungicides. Preventing disease outbreak is a more cost-efficient and ecologically sustainable option. Fungicides and their application are not only monetary factors, but may also be overcome by resistant fungal strains and have less of a negative impact on the environment which can lead to even higher consequential expenses (Bolton et al., 2013).

The hyperspectral signature of the leaf, which reflects its physiological status, varies in response to a number of imposed biotic or abiotic stresses (Blackburn, 1998, 2007). Hyperspectral imaging has the advantage of being a non-invasive assay and can be applied to follow a dynamic process. The method relies on the capture of the reflectance spectrum containing specific absorption properties of the subject in response to exposure to broadband illumination, typically in the visible (400–700 nm), near-infrared (700–1100 nm), and short-wave infrared range (1100–2500 nm; Mutka and Bart, 2014). The hyperspectral signatures are acquired in a pixelwise manner, whereby each pixel recorded from a hyperspectral line camera is containing an individual spectral profile. Artificial neural network computing (ANN) is a machine learning technique that when applied to hyperspectral signatures can be utilized to predict classes, e.g., healthy or diseased plant (Backhaus et al., 2011). The approach has been used to evaluate the physiological and disease status of plants both in a greenhouse setting and conducting airborne sensing of field crops (Lelong et al., 1998; Asner et al., 2007; Gowen et al., 2007; Backhaus et al., 2011; Carvalho et al., 2012). Hyperspectral images combine three data dimensions whereby two are of spatial nature and one of spectral, giving rise to a wealth of information to investigate, e.g., plant phenotypes (Fiorani and Schurr, 2013).

The availability of a non-invasive, highly predictive and early detection of this disease is attractive, as it will allow improvements in crop management and in the selection efficiency toward breeding for disease resistance (Mutka and Bart, 2014). Hyperspectral imaging has been demonstrated to be feasible for the detection of developed symptoms and identification of several sugar beet leaf diseases, including *Cercospora* leaf spot (Mahlein et al., 2010, 2012). However, the transition from simply detecting symptoms on the leaf surface caused by the presence of the fungus to presymptomatic (without visible symptoms) infection recognition requires both a more advanced mathematical data analysis and the general existence of a pathogen-specific defense reaction of the host plant. Here, the aim was to develop the means to non-invasively detect presymptomatic *Cercospora* leaf spot disease in selected sugar beet cultivars which differed from one another with respect to disease susceptibility. The intention was firstly to provide a proof-of-concept for a screening method usable within the context of a breeding program targeted at improving the level of resistance to *Cercospora* leaf spot; and secondly, to characterize which metabolic pathway(s) were affected early in the process of infection. A focus was set on phenolic compounds, which are known to contribute to defense either as preformed or as induced by pathogen. Hyperspectral and metabolic data correlated well and facilitated filtering for relevant metabolites.

## Materials and Methods

### Plant Material

Three sugar beet cultivars (STR1, STR2, and STR3), differing in their level of susceptibility to *Cercospora* leaf spot (STR1 resistant, STR2 tolerant, and STR3 susceptible), were grown in pots in a greenhouse (Strube, Schlanstedt, Germany) held at 60% relative humidity, 18/22°C (day/night) and a 16 h photoperiod. The cultivars are genetically diverse. The plants were kept fully watered and fertilized with 0.1% N/P/K solution once a week. The experiments were initiated after about 8 weeks, at which point the plants had reached growth stage 16 (Meier et al., 1993).

### Stable Isotope Labeling with ^15^N

Sugar beet seed was germinated on moist filter paper and kept in the dark. After 7 days, seedlings were transferred to half strength Hoagland’s media (Hoagland and Arnon, 1950) and grown hydroponically for 6 weeks at 20/18°C (day/night) under a 16 h photoperiod provided by 300 μmol m^-2^ s^-1^ light. Half of the seedlings were subjected to medium containing ^15^NH_4_^15^NO_3_ (atom % 98, Campro Scientific GmbH, Berlin, Germany). Leaves of 6 weeks old plants were harvested.

### Inoculation with *C. beticola*

The isolate Holtensen 2011 (obtained from Mark Varrelmann, IfZ Göttingen) of *C. beticola* was used for the experiments. *C. beticola* isolate was multiplied by culturing them on agar-solidified V8 medium at 25°C under natural daylight for 2 weeks. Mycelia and spores were scraped off the plate and suspended in sterile water to produce an inoculum containing 50,000 infectious units per mL, which was sprayed uniformly over the test plants, and the spraying was repeated 2 h later. After inoculation a foil tunnel was established to obtain a humidity of ≥95%. The lights were switched off for the next 3 days, after which the foil covering was removed and the growing conditions were set to deliver 28/20°C (day/night) and a 14 h photoperiod.

### Quantification of *C. beticola* Biomass

Fungal biomass was quantified according to De Coninck et al. (2012) with modifications. DNA was extracted from 100 mg frozen leaf material or plate-grown fungal mycel using DNeasy Plant Mini Kit (50) (Qiagen, Venlo, Netherlands) according to the manufacturer’s instructions. Real time quantitative PCR (qRT-PCR) was performed with a LightCycler^®^ 480 (Roche Diagnostics, Risch, Switzerland) using 40 cycles (95°C 10 s, 60°C 30 s). In contrast to De Coninck et al. (2012) CercoCal1 and SbEc1 were amplified in separate reactions, each containing 5 μl SsoAdvanced SYBR Green Supermix (Bio-Rad, Hercules, CA, USA), 2 μl DNA extract (10 ng/μl), 1.5 μl primermix (2 pmol/μl) and 1.5 μl water. *C. beticola* infestation was calculated by two methods, the comparative method (Δct = ct_CercoCal1_ – ct_SbEc1_) or by utilizing a calibration curve of fungal DNA (Supplementary Figure S1). Statistics including Student’s *t*-test were performed using Microsoft Excel^1^ software.

### Hyperspectral Image Acquisition and Pre-processing

Young sugar beet leaves where placed on a low reflective base, which was moved on a translation stage mounted 1 m below a HySpex SWIR-320m-e line camera (Norsk Elektro Optikk A/S, Skedsmokorset, Norway). To calibrate the set-up for relative reflectance, a standard optical PTFE (polytetrafluoroethylene) card was included in each image. The spectra captured in the infra-red range (970–2,500 nm) had a spatial resolution of 6 nm and yielded a 256 dimensional spectral vector per pixel. The camera line’s spatial resolution was 320 pixel, and its maximum frame rate was 100 fps. Radiometric calibration was performed using the vendor’s software package. Image processing was performed using a workflow system implemented in Matlab (The MathWorks, Inc., Natick, MA, USA). The standard calibration pad is automatically detected within the image and extracted. Each spectrum was referenced against the calibration pad mean spectra to obtain a set of relative reflectance spectra per pixel. Next, the spectral signatures were grouped into five clusters on the basis of their similarity, as measured by Euclidean distance. Grouping was performed by Neural Gas Clustering (Martinetz and Schulten, 1991). Each pixel belongs to one particular cluster and these clusters were used to obtain proper image segmentation. Clusters representing leaf material were selected automatically by means of reference spectra manually selected in one image. The pre-processing steps for extracting pixel spectra representing plant material are illustrated in Supplementary Figure S2.

### Machine Learning for Spectral Data Analysis

A machine learning approach was followed to generate a classification system to enable the pre-symptomatic detection of *Cercospora* leaf spot. As a first step, the machine learning approach was tested for its capability to differentiate the cultivars and in the second step to differentiate the infection status per cultivar. An ANN was trained to convert the input (spectral information) into category (infection state, cultivar). During the training phase, the parameters were adjusted to reduce the prediction error as much as possible. LDA was performed in order to search for a linear combination of features capable to discriminate between two or more classes of objects or events. Note that the number of features or dimensions resulting from an LDA is N-1 where N is the number of classes. The classification was based on both a single model Radial-Basis Function (RBF) Network (Moody and Darken, 1989; Backhaus et al., 2012) and on a multi-model approach; for the latter, the first layer assumed an RBF model with various metrics to calculate spectral similarities, after which a fusion RBF model was applied to combine the output of all the single models (Knauer et al., 2014). The models were validated using a standard five-fold cross validation design, in which the data were assigned to five equally sized partitions, four of which were used for training and the fifth for validation. Mean classification rates across test samples are reported.

Systematic dependencies of metabolite data and spectral data were revealed by applying an RBF Network to map spectral reflectance input data onto metabolite output data. Differentially abundant metabolites were ordered on the basis of their observed FCs. The RBF Network was trained following a “leave-one-out” scheme, in which the data captured from one replicate or leaf number was held in reserve until the model had been derived from the remaining data. The predicted peak intensity for the reserve sample was then used to calculate a coefficient of determination (R^2^), which varies from 1 (perfect prediction) to 0 (zero prediction).

### Extraction of Semi-Polar Compounds

Leaf material was harvested, analyzed by the hyperspectral imaging system and snap- frozen in liquid nitrogen. The frozen leaf was then lyophilized and milled to a powder, of which a 15 mg aliquot was extracted in 900 μL aqueous methanol (75% v/v) with formic acid (0.1% v/v). Cell disruption was promoted by the inclusion of 1.0–1.2 mm diameter zirconium beads (58% ZrO_2_) (Muehlmeier, Baernau, Germany). The samples were centrifuged (27,500 × *g*, 10 min, 4°C), and 20 μL 0.1% formic acid was added to an 80 μL aliquot of the resulting supernatant. After a second centrifugation (27,500 × *g*, 5 min, 4°C), the supernatant was subjected to liquid chromatography–mass spectrometry(LC–MS).

### Metabolite Profiling via (U)HPLC-UV-ESI-MS

Chromatographic separation and UV detection were carried out using a Dionex Ultimate 3000 RSLC system (Thermo Fisher Scientific, Inc., Waltham, MA, USA). The LC system was fitted with an Acquity UPLC^®^ BEH Phenyl column (130 Å, 1.7 μm 2.1 mm × 100 mm), in combination with an Acquity UPLC BEH Phenyl VanGuard pre-Column (130 Å, 1.7 μm, 2.1 mm × 5 mm). The temperature of the column was maintained at 35°C. The solvent was administered at a flow rate of 500 μL/min, according to the following protocol: 0–1 min: 5% solvent B, 1–10 min: 5–40% solvent B, 10–11.5 min: 40–97% solvent B, 11.5–13 min: 97% solvent B, 13–13.5 min: 97–5% solvent B; 13.5–17 min: 5% solvent B. Solvent B was acetonitrile (Chemsolute, Renningen, Germany)/0.1% (v/v) formic acid (J. T. Baker, Deventer, Netherlands), and solvent A was 18 mΩ water (Merck, Darmstadt, Germany)/0.1% (v/v) formic acid. The mass spectrometry data were acquired by using a Bruker maXis Impact device (Bremen, Germany) coupled to the LC system. Electrospray ionization (ESI) was implemented in positive mode at 200°C dry temperature, three bar nebulizer, 4000 V capillary voltage and a dry gas flow of 8 L/min. The MS settings were adjusted to cope with small molecules (50–1,000 *m/z*), a hexapole radio frequency (RF) voltage of 40 V peak-to-peak (Vpp), a collision energy of 10 V, a funnel 1 RF of 300 Vpp, a funnel 2 RF of 300 Vpp, a prepulse storage time of 5 μs, a transfer time of 50 μs and a collision cell RF of 500 Vpp. The routine was run in MS^2^ scan modes: MS/MS (auto) and MRM.

### Identification of Metabolites

Liquid chromatography–mass spectrometry data analysis was performed using Compass Data Analysis 4.1, Compass Profile Analysis 2.1 and various Compass utility tools offered by Bruker. Data pre-processing for statistical analysis started with an internal calibration for mass accuracy using ‘quadratic + High Precision Calibration (HPC)’ mode, which was performed for each injection with calibration solution containing 10 mM sodium formate. After calibration, the peak finding algorithm ‘Find Molecular Features’ (FMFs) was applied to extract relevant *m/z* signals. The FMF data were normalized aligned and used to calculate a ‘bucket table’ which plots the mass retention time pairs against the intensities of the individual samples. Principal component analyses (PCA) was utilized to reduce the multivariate data set to its leading features. The Pareto scaling algorithm was applied with a confidence level of 95% to calculate the PCAs. Samples were grouped according to their treatment condition, and compared using the Student’s *t*-test to identify differentially abundant metabolites. The selection criteria were a FC of at least 1.5 and a FWER *P*-value of less than 0.05 (Bonferroni-corrected). Sum formulae were calculated by SmartFormula software, based on mass and isotope pattern information. Stable isotope labeling with ^15^N was used to confirm the identification of N-containing molecules. Following MS^2^ analysis, fragmentation patterns were queried against the massbank database^2^ and *in silico* verification was performed using MetFrag^3^ and MetFusion^4^ (Gerlich and Neumann, 2013) routines. When available, authentic standards were used to confirm the annotation. In-source fragmentation products were sorted manually on the basis of MS^2^ data and elution time. (U)HPLC-UV data were processed and monitored at 280 nm, and the outputs analyzed using Compass Data Analysis 4.1 and Microsoft Excel^5^ software.

## Results

### Comparison of *Cercospora beticola* Infestation in Sugar Beet Genotypes

**FIGURE 1:**
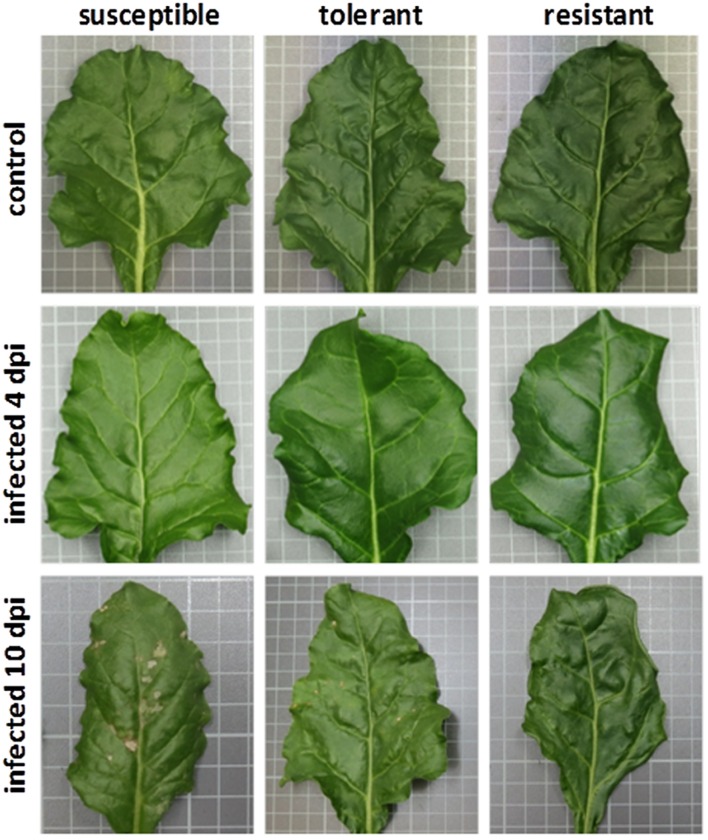
**The leaves of non-infected and infected plants**. Neither type shows any visible disease symptoms when observed 4 days post-infection (dpi). At 10 dpi leaves of the susceptible genotype showed numerous large necrotic lesions. The tolerant genotype exhibits smaller lesions. No necrosis was detected on leaves of the resistant genotype.

**FIGURE 2:**
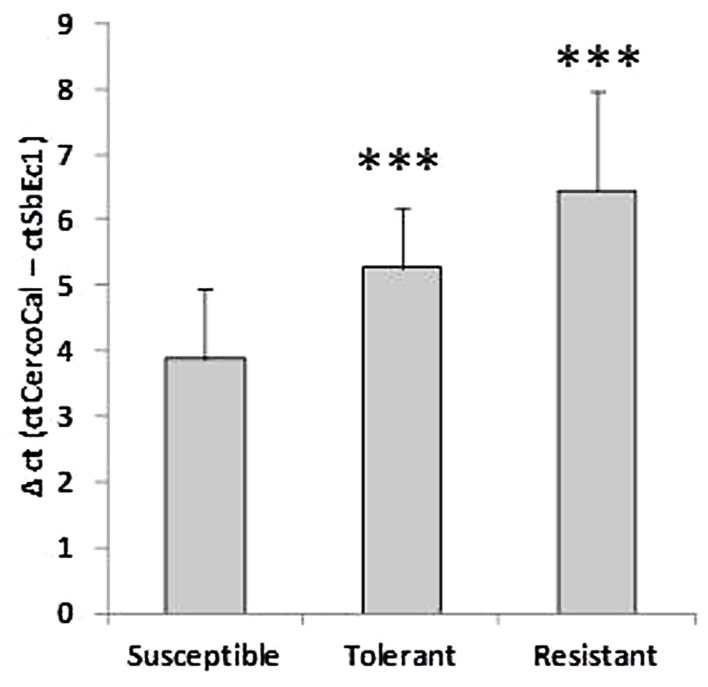
**Comparative analysis of the fungal biomass in three genotypes**. Values for plant DNA (ct of SbEc1) were subtracted from values for *Cercospora beticola* DNA (ct of *Cercospora* calmodulin) to determine a relative value for fungal biomass (Δct). The lower the Δct value the higher the amount of fungal biomass. Mean values were calculated from three independent experiments containing at least six biological replicates per condition (*n* = 18). ^∗∗∗^*P* < 0.001.

At 10 dpi the selected sugar beet genotypes showed contrasting numbers of *C. beticola* induced necrotic lesions (**Figure 1**). The susceptible genotype displayed numerous large necrotic spots, while the size of lesions was lower in the tolerant genotype. The resistant genotype showed no lesions. The susceptibility to *Cercospora* leaf spot was quantified by qRT- PCR. Comparison of absolute quantification of fungal biomass to relative quantification (Δct is determined by subtraction of ct values of an endogenous plant target from ct values from fungal calmodulin) showed high correlation *R*^2^ = 0.97 (Supplementary Figure S3). Therefore, both methods were equally suitable for quantification. **Figure 2** shows the mean values for relative quantification obtained from three independent experiments including each six biological replicates per condition (*n* = 6 per experiment). The higher the Δct value, the less fungal biomass was detected compared to plant biomass (De Coninck et al., 2012). Fungal biomass was significantly different between all genotypes, whereby leaves of the susceptible genotype contained the highest amount of fungal biomass.

### Cultivar-Specific Differences in the Hyperspectral Signatures and Metabolite Profiles

**FIGURE 3:**
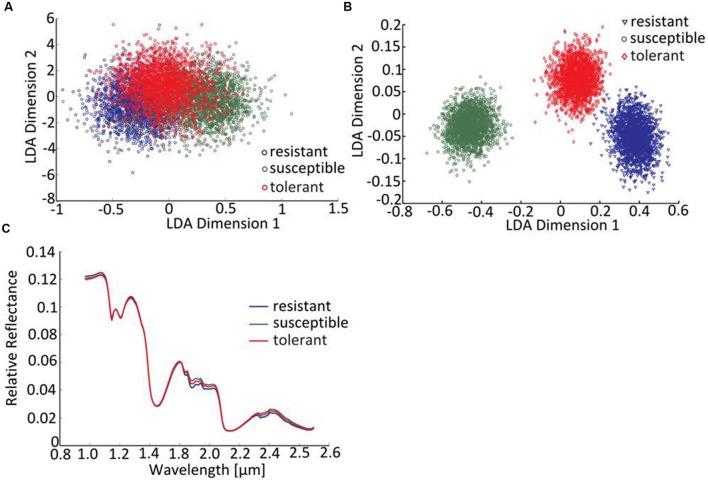
**Distinguishing the three cultivars by hyperspectral imaging**. **(A)** The LDA of reflectance spectra (single model), **(B)** the LDA of reflectance spectra (first layer of multi-model classification neural network). **(C)** The mean reflectance spectra for each cultivar (plants not exposed to *C. beticola*).

**FIGURE 4:**
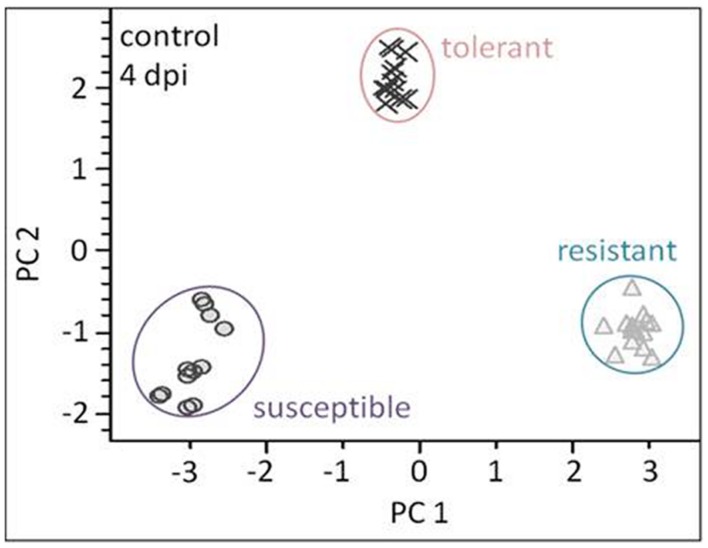
**Liquid chromatography–mass spectrometry (LC–MS) based metabolite profiling of the three cultivars (plants not exposed to *C. beticola*) as analyzed by PCA**. The metabolite profiles of all genotypes show high separation. Each symbol represents one analysis (two technical replicates of 6–7 probes from individual plants).

**FIGURE 5:**
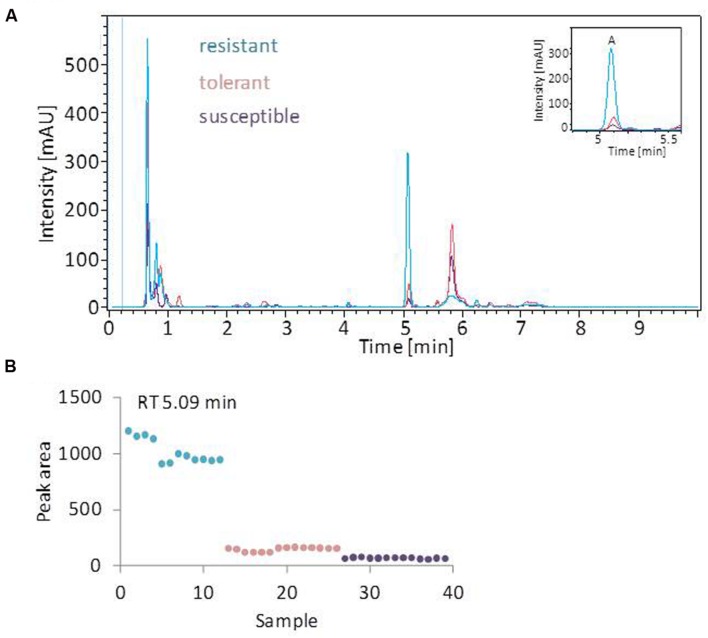
**UV profiling of leaf extracts**. **(A)** Overlay of typical (U)HPLC-UV chromatograms obtained from healthy leaves. The profiles of the tolerant and susceptible cultivar are highly similar. The most prominent peak (peak A) is most strongly represented in the resistant cultivar. **(B)** The quantification of *o*-glucosylvitexin (corresponding to peak A), based on the UV-peak area. Each symbol represents one analysis (two technical replicates of 6–7 probes from individual plants).

The linear discriminant analysis (LDA) of the hyperspectral reflectance signature was not capable of discriminating between the host cultivars when a single model approach was applied (**Figure 3A**). A better level of discrimination, which is also reflected in the higher prediction accuracy, was achieved by applying the multi model and visualizing its first layer in an LDA plot (**Figure 3B**). The reflectance signature of the cultivars varied mainly between 1,600–1,850 and 2,150–2,400 nm (**Figure 3C**), and there was a high level of dissimilarity between the signatures of the susceptible and the resistant cultivar. The LC–MS analysis of the semi-polar metabolites revealed a cultivar-specific pattern in non-inoculated plants (**Figure 4**). PCA were calculated using the intensities of mass retention time pairs (from here on named ‘features’). The PCAs generated a distinct cultivar-specific clustering, in which the first principal component accounted for 52.7% of the explained variance and the second component for 25.5%. The (U)HPLC-UV-chromatograms revealed a higher level of similarity between the tolerant and the susceptible cultivar than between either of them and the resistant one (**Figure 5A**). The most prominent difference between the three genotypes was the peak associated with an *m/z* of 595.17, identified by MS/MS^2^ analysis as glucosylvitexin (Supplementary Figure S4), a compound known to be present in the leaves of beet (Isayenkova et al., 2006). The intensity of this peak could not be used for quantification as the concentration differences between the cultivars were outside the dynamic range of the mass spectrometry device. Instead, its UV signal was used for quantification. The intensity differences were estimated at 15 fold between the resistant cultivar and the susceptible one, and seven fold between the resistant cultivar and the tolerant one (**Figure 5B**).

### Hyperspectral Imaging Enables the Detection of Presymptomatic *Cercospora* Leaf Spot and Is Predictive of Metabolite Status

**FIGURE 6:**
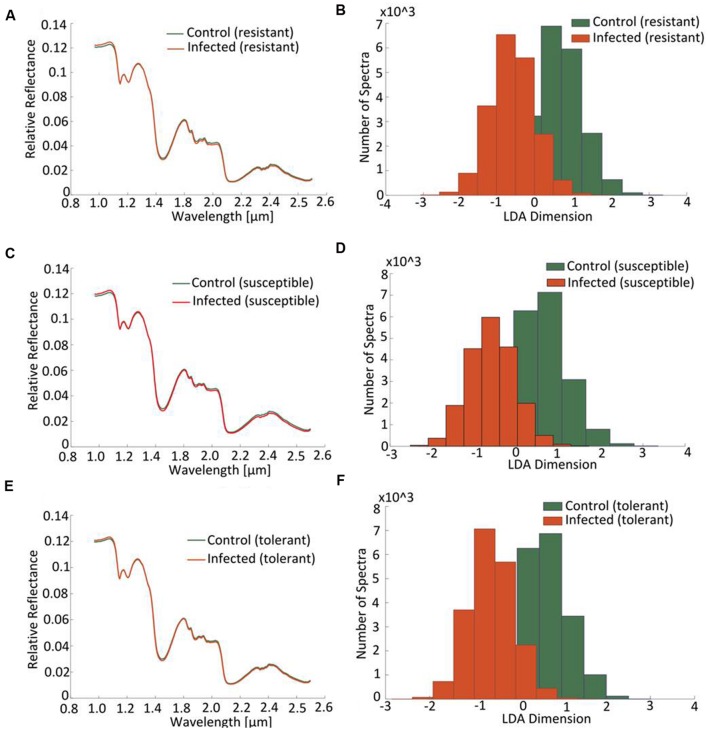
**Distinction between cultivars obtained by hyperspectral imaging of leaves sampled at a presymptomatic stage (4 dpi) of a *Cercospora* leaf spot disease**. Comparison of the normalized relative reflectance spectra between non-infected and infected plants, the profiles differ only marginally from one another **(A,C,E)**. The mapping of the spectral information onto one dimension resulting from a multi-model LDA depicts the distribution of pixels belonging to “non-infected” or “infected”: the less the extent of overlap, the more well-separated are the groups **(B,D,F)**.

At the early stage of infection [4 days post-inoculation (dpi)], the hyperspectral signatures of the leaves differed only slightly from those of the non-infected leaves (**Figure 6**, left side). However, these subtle differences were still sufficient to perform a robust classification utilizing the advanced concept of multi-models. The outcomes of the single and multi-model analyses are shown in **Table 1**. Mean accuracy values and its standard deviation across cross-validations for the test data are reported. The LDA of the first layer of the multi-model approach depicted in **Figure 6** (right hand side) shows a clear separation between the non-infected and infected plants for each of the three cultivars. No visible symptoms of infection were apparent at 4 dpi, but the hyperspectral signatures allowed an unequivocal classification of their infection status.

**Table 1:** The classification accuracy achieved by models derived from hyperspectral data collected from a presymptomatic sugar beet infected with *C. beticola*.

Method	Resistant		Tolerant		Susceptible	
	Mean accuracy	Standard accuracy	Mean accuracy	Standard accuracy	Mean accuracy	Standard accuracy
Single model	78.4%	1.4%	74.9%	2.50%	69.4%	2.9%
Multi model	99.9%	0.1%	99.6%	0.2%	98.5%	0.2%

*The mean accuracy and associated standard deviation obtained from a fivefold cross validation of infected vs. non-infected leaf were based on single pixel spectra.(A 100% accuracy score is realized when each pixel is assigned to the correct class.) Two different methods have been used, a single model approach with one Neural Network and a multi-model approach, in which an ensemble of classifiers is used to predict the respective class for each pixels. The rate of successful classification was increased by using a multi-model approach*.

The coefficients of determination (*R*^2^) for the set of selected metabolites are shown in **Table 2**. Based on the reflection spectrum, the neural network was able to predict the below-peak areas with a high level of precision (*R*^2^ values up to 0.94), demonstrating a systematic dependency between the hyperspectral signature and the metabolite status of the plant. The single model approach was moderately accurate, with the rate of correct classification lying from 70 to 80% (**Table 1**). Implementing the multi model raised this rate to >98.5%. The resistant cultivar was classified with 99.9% accuracy.

**Table 2:** Differentially abundant metabolites in young sugar beet leaves sampled 4 days after infection with *C. beticola*.

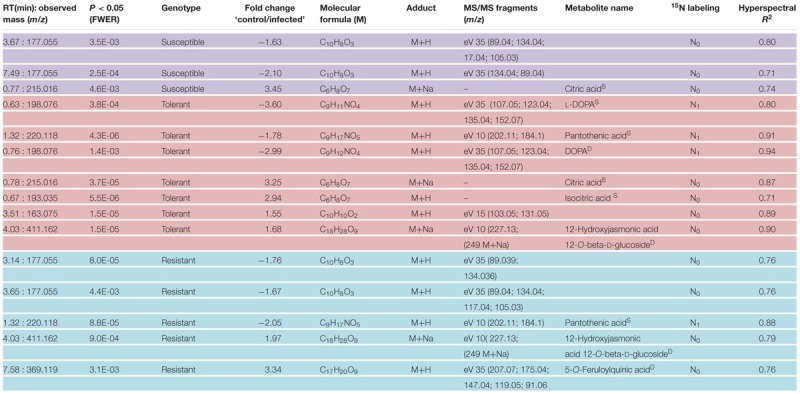

*The metabolites were selected on the basis of their showing a high correlation (*R*^2^ ≥ 0.7) with the hyperspectral data. Stable isotope labeling with ^*15*^N was used to confirm the molecular formulae of nitrogenous compounds. Annotation was based on comparison with authentic reference standards (level 1) or databases (level 2) (Sumner et al., 2007). More details are given in Supplementary Table S1. ^S^Identified based on authentic reference standard; ^D^annotation based on database search*.

### Metabolite Profiles Respond Rapidly to Pathogen Infection

**FIGURE 7:**
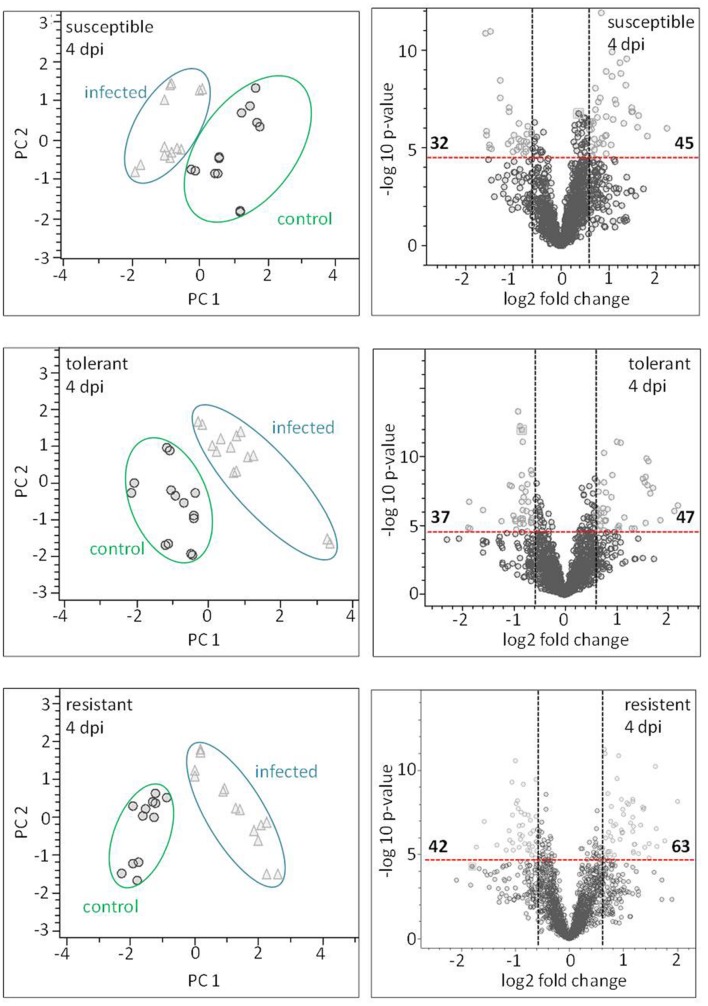
**Metabolite profiling of cultivars: leaves sampled at a presymptomatic stage of a *Cercospora* leaf spot disease**. The LC–MS data-based PCA reveals some clustering of metabolite profiles. Each symbol represents one analysis (two technical replicates of 6–7 probes from individual plants). The most well-separated host was the resistant cultivar, suggesting that it experiences the most profound reprogramming as a result of the fungal infection. The volcano plots depict significantly different features in all three cultivars in the contrast “non-infected” vs. “infected” (light gray). The thresholds were indicated with a red dashed line for the corrected (FWER; *P* ≤ 0.05), and a black dashed line for FC ≥ 1.5.

Principal component analysis was employed to differentiate between the metabolic profiles of control and infected plants. The PCAs scores plots demonstrate that a clear discrimination could be drawn between the metabolite profiles produced by the non-infected and infected plants for each cultivar already at just 4 dpi (**Figure 7** left hand side). The higher the levels of resistance, the more distinct were the profiles. For the susceptible cultivar the first principal component (PC) accounted for 25.5% of the variance and the second PC for 20.4%. The equivalents for the contrast between non-infected and infected in the resistant and the tolerant cultivar were, PC1 36.6%, PC2 14.0% and PC1 26.8% PC2 21.9% explained variance, respectively.

### The Identification of Disease-Responsive Metabolites

Based on the chosen significance criteria [family wise error rate (FWER) *P* ≤ 0.05 and fold change (FC) ≥| 1.5| ], inoculation with the pathogen altered 105 features in the resistant cultivar, 84 in the tolerant cultivar and 77 in the susceptible cultivar (**Figure 7**, right hand side). The features showing the highest FC were largely, but not exclusively, cultivar-specific. The tolerant and the resistant cultivars exhibited the highest degree of similarity (36 features), and the susceptible and resistant ones the lowest (22 features; Supplementary Figure S5). Features associated with an *R*^2^ value ≥ 0.7 were considered to be highly correlated to the hyperspectral signatures and were annotated (**Table 2**). Accurate mass and isotopic pattern led in most cases to a molecular formula for the compounds. A full list including in-source products is provided in Supplementary Table S1. In the compatible (susceptible) interaction, the abundance of citric acid was decreased (FC = 3.45) and that of two compounds with the molecular formula C_10_H_8_O_3_ was increased (-1.63, -2.10). In both the tolerant and resistant cultivar, the concentration of pantothenic acid was boosted by the presence of the fungus, while that of 12-hydroxyjasmonic acid 12-*O*-β-D-glucoside (12-*O*-Glc-JA) was reduced (-1.68, -1.97). In the infected leaf of the tolerant (but not the resistant) cultivar, the level of DOPA was significantly raised; while in the resistant (but not the tolerant) cultivar, the level of 5-*O*-feruloylquinic acid was reduced (3.34).

## Discussion

In the present study, selected sugar beet genotypes differing in their degrees of susceptibility to *Cercospora* leaf spot could clearly be distinguished by metabolite profiling (invasively) and by hyperspectral imaging (non-invasively). Furthermore the feasibility of non-invasive highly accurate detection of *C. beticola* infection of sugar beet plants using hyperspectral imaging was shown. As discussed in detail below, the nature of the changes to the leaf metabolome induced presymptomatically by *C. beticola* infection may be informative regarding the molecular difference between a compatible and an incompatible host–pathogen interaction. Finally, the experiments set out to identify candidate metabolites associated with either constitutive or induced resistance to *Cercospora* leaf spot.

### The Hyperspectral Signatures Differentiate Genotypes and Suggests the Existence of Pre-formed Defense Compounds

In the absence of the pathogen, the three cultivars differed both with respect to their metabolome and their hyperspectral signature, which shows constitutive differently abundant compounds, some of which are likely to be involved in defense. In particular, glucosylvitexin was highly abundant in the resistant cultivar. The glycosylated form of vitexin may be more readily stored in the cell than the active form and hydrolyzed as a reaction to environmental cues. Vitexin has been implicated in the resistance of cucumber against powdery mildew (McNally et al., 2003), and has also been associated with the biotic stress response in certain cereal species (Balmer et al., 2013). The photosensitizing properties of cercosporin, the toxin responsible for the pathogenicity of *Cercospora* spp. (Upchurch et al., 1991; Daub and Ehrenshaft, 2000; Staerkel et al., 2013), lead to the formation of toxic singlet oxygen molecules (^1^O_2_) and superoxides (⋅O_2_^-^) (Daub and Hangarter, 1983). Thus, the ROS scavenging properties of vitexin could be advantageous for preventing cercosporin induced cell damage (Shibano et al., 2008). Constitutive defense offers several advantages to the host: it serves as a means to circumvent reduction of defense responses due to fungal effectors, and helps to limit the growth of the fungus by avoiding the time lag involved between the initial infection and the metabolic reprogramming required to mount an induced defense response. On the negative side, in the absence of pathogen pressure, it imposes a metabolic cost on the host, which is reflected in a reduced yield potential.

### Quantification of *Cercospora* Leaf Spot

Differences in susceptibility to *Cercospora* leaf spot were visually detected and quantified by qPCR in three selected genotypes. Necrotic lesions were observed on leaves of the susceptible (numerous, large partly merging) and tolerant (smaller, separate) genotype. Despite the detection of fungal biomass in all genotypes there were no spots visible on leaves belonging to the resistant genotype. Feindt et al. (1981) described equal sporulation behavior and epidermal growth of *C. beticola* on susceptible and resistant cultivars. Therefore, the fungal DNA detected in the resistant genotype could be originated from initial epidermal hyphal growth. Possibly, the lesion development and the collapse of plant cells is delayed or inhibited by a strong defense response in the resistant genotype. When plant cells are intact the nutrient availability in the intercellular space is poor, which might lead to termination of hyphal growth due to starvation. With this qPCR method presence but not viability of fungal DNA could be assessed.

### Hyperspectral Imaging Enables the Early Diagnosis of *Cercospora* Leaf Spot Infection

The hyperspectral imaging technique has demonstrated its ability to diagnose *Cercospora* leaf spot disease presymptomatically with the highest reported classification accuracy (98.5–99.9%) by hyperspectral imaging (Rumpf et al., 2010; Mahlein et al., 2012), representing thereby a significant advance toward the automation of early generation screening in a resistance breeding program. It is apparent that hyperspectral signatures are genotypically variable, so an important validation step will be to trial the method on a segregating population.

In other contexts, hyperspectral imaging has been demonstrated to be suitable for the estimation of metabolites, for instance the aflatoxin concentration on corn kernels was successfully associated with hyperspectral imaging data (Yao et al., 2010). In addition, predictive models based on reflectance signatures could predict cardenolide concentration in milkweed upon wounding (Couture et al., 2013). On a wider level, the technology has been applied to assess foliar polyphenol and nitrogen content across entire landscapes (Skidmore et al., 2010).

### Presymptomatic Metabolic Defense Response Was Detected

This is the first study performing metabolite profiling on the early response reaction of sugar beet cultivars with differing degree of susceptibility. Some candidate metabolites correlated positively with hyperspectral signatures and possibly contribute to the spectral reflectance. These more relevant candidates were further annotated. A major challenge in the field of metabolomics is the annotation of compounds of secondary metabolism because authentic standards are not always available and there is limited information in databases concerning MS and MS^2^ spectra. In-source fragmentation is another major issue which can lead to fragments that are mistaken as cellular metabolites (Xu et al., 2015). Time consuming manual data inspection is often required, thus filtering candidates for relevance is helpful.

### Metabolic Response to *Cercospora* Leaf Spot during Incompatible Interaction

The metabolomics data has provided novel insights to incompatible plant–pathogen interaction and is being preliminary discussed here but functional analysis has to follow regardless. The metabolites were identified with authentic reference substances, if available, or annotated with MetFusion based on exact mass and fragmentation pattern. The leaf content of *pantothenic acid* was markedly increased in both the tolerant and the resistant cultivar following their infection by *C. beticola*. This compound, also referred to as vitamin B5, functions as a precursor of coenzyme A (CoA; Smith et al., 2007), which is involved in a wide range of biological processes, including the TCA cycle and both fatty acid and phenylpropanoid metabolism. A scan of the literature suggests that this is the first documented instance of pantothenic acid being associated with the biotic stress response. Many of the (pro-) vitamins (A, B1, B6, B9, C, E, K1) have been associated with antioxidative potential (Asensi-Fabado and Munné-Bosch, 2010), and the structure of pantothenic acid includes three free hydroxyl groups with potential antioxidative activity; thus it may be that by accumulating pantothenic acid, the sugar beet plant gives itself a measure of protection against cercosporin-induced oxidative stress. Vitamin B6 has demonstrated some capacity within *Cercospora* to inhibit cercosporin autotoxicity (Bilski et al., 2008), and is known as a protectant against photo-oxidative stress (Chen and Xiong, 2005; Titiz et al., 2006; Havaux et al., 2009). Further experiments are of need to elucidate the role of vitamin B5 in stress response whether it is playing an active part or it is an accumulating intermediate.

The molecule *12-O-Glc-JA*, initially termed tuberonic acid glucoside (Yoshihara et al., 1989), was less abundant in the infected leaf of both the tolerant and the resistant cultivar than in that of the susceptible one.

Glycosylation is discussed to be a modification of the bioactive aglycon that enables transport and/or storage (Jones and Vogt, 2001). Plant hormones are also subjected to it, for instance increased glycosylation was shown to affect abscisic acid (ABA) homeostasis (Priest et al., 2006). As a consequence to environmental stimulus enzymatic hydrolysis of storage compounds releases the physiologically active form. The observed reduction in 12-*O*-Glc-JA content upon infection suggests its conversion by deglycosylation. In rice, an enzyme (OsTAGG1) has been purified which is capable of deglycosylating 12-*O*-Glc-JA to form the physiologically active form 12-hydroxyjasmonic acid (12-OH-JA; Wakuta et al., 2010). 12-*O*-Glc-JA is reportedly synthesized from the phytohormone jasmonic acid (JA), which has variety of functions in biotic stress defense; for example, it regulates the production of phenylpropanoids (lignins, flavonoids, and other antioxidants; Gundlach et al., 1992). Storage forms might provide a safety net for plants when *de novo* synthesis is inhibited due to fungal effector molecules disturbing pathogen defense. 12-*O*-Glc-JAs can be transported to underground parts and act as a signaling molecule that may mediate changes of source-sink relationship upon pathogen attack (Yoshihara et al., 1996; Seto et al., 2009). JAs have various functions in defense and development whereby defense is prioritized over growth, for instance root growth inhibition (Yang et al., 2012; Wasternack and Hause, 2013). Activation of JA signaling cascade in the tolerant and resistant sugar beet genotypes suggest a more efficient recognition of *C. beticola* by MAMPs/PAMPs and subsequent triggering of defense responses.

The leaf content of *5-O-feruloylquinic acid* was lowered in the resistant cultivar upon infection. This compound belongs to the chlorogenic acids (Clifford, 1999), which act as important intermediates in lignin synthesis (Vanholme et al., 2010). So a reduced level of 5-*O*-feruloylquinic acid implies an increased rate of lignin synthesis. Lignin is of prime importance for the physical strength of the cell wall; in cotton, it has been shown that resistance to the pathogen *Verticilium dahliae* is associated with an increased level of cell wall lignification (Xu et al., 2011).

The tolerant sugar beet cultivar responded to the presence of *C. beticola* by accumulating the molecule *L-DOPA*, a precursor of dopamine, thought to play a role in the host’s resistance to *Cercospora* leaf spot (Harrison et al., 1970). In addition, L-DOPA and dopamine are both strong antioxidants, so could help in the scavenging of the reactive oxygen species induced by the action of cercosporin (Kanazawa and Sakakibara, 2000; Gülçin, 2007).

In summary, genotypes could be distinguished based on their hyperspectral signature and on their metabolic profiles. Whether metabolites contribute to the hyperspectral signature or act as separate markers is unclear. Further experiments to investigate the contribution of phenolic compounds to the hyperspectral signature in sugar beet leaves subjected to modified light conditions are underway.

The study represents a successful proof-of-concept for an effective and efficient screening system for presymptomatic identification of *Cercospora* leaf spot. Compared to the widely used visual disease assessment, quantification of disease severity by hyperspectral imaging provides the advantage of standardized and objective measurement. Further, phenotyping by hyperspectral imaging is amenable to automation, and high-throughput analysis is possible after the initial establishment is realized. As a result, the need for greenhouse space, labor and time can be reduced. Hand-held devices capable of hyperspectral phenotyping under field conditions are currently under development and could potentially improve effective fungicide application. The characterization of the metabolomes of the contrasting cultivars in response to *C. beticola* infection has provided interesting new candidates for the components of the defense response. The biological role of these compounds in the context of the host–pathogen interaction remains to be characterized; however, in the meantime, they can be used as informative candidates associated with resistance in beet.

## Author Contributions

Contributions to conception and design of this study: NA, AB, SF, US, and H-PM; Participation in drafting and revising of the manuscript: NA, AB, SD, SF, US, and H-PM; Experiments and data acquisition: plant cultivation and pathogen inoculation: SF; Hyperspectral imaging: AB; Metabolomics study: NA.

## Conflict of Interest Statement

The authors declare that the research was conducted in the absence of any commercial or financial relationships that could be construed as a potential conflict of interest.

## Footnotes

1 https://products.office.com/en-gb/excel

2 www.massbank.jp

3 http://msbi.ipb-halle.de/MetFrag/

4 http://msbi.ipb-halle.de/MetFusion/

5 https://products.office.com/en-gb/excel
