# Supplementary material for: Non-invasive Presymptomatic Detection of Cercospora beticola Infection and Identification of Early Metabolic Responses in Sugar Beet
Source: Front Plant Sci. 2016 Sep 22;7:1377. doi: 10.3389/fpls.2016.01377 (PMC5031787; doi:10.3389/fpls.2016.01377)
Supplement: Supplementary file 1 [file Data_Sheet_1.DOCX]

Supplementary Material

**Noninvasive presymptomatic detection of *Cercospora beticola* infection and identification of early metabolic responses in sugar beet**

Nadja Arens, Andreas Backhaus, Stefanie Döll, Sandra Fischer, Udo Seiffert, Hans-Peter Mock*

*** Correspondence:** Hans-Peter Mock: mock@ipk-gatersleben.de

**
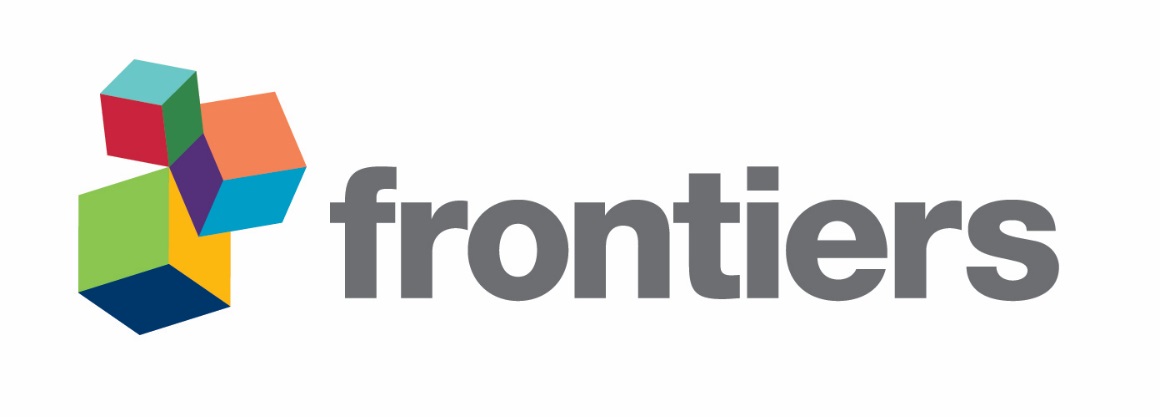
**

**Supplementary**

**Supplementary Figure S1 Dilution series of C. beticola DNA for calibration curve**

For absolute quantification of fungal biomass DNA was isolated from plate grown C. beticola, diluted and analyzed by qPCR to generate a reference curve.

**Supplementary Figure 2.** Hyperspectral image acquisition and object extraction. Hyperspectral image acquisition and object extraction – (a) Plant material is placed on a low reflective rubber material and recorded with a PTFE (polytetrafluoroethylene) calibration standard; (b) a cluster algorithm performs a grouping of spectral information due to some similarity criteria, grouping is displayed with different color overlays; (c) calibration standard is automatically detected using image processing / object recognition; (d) the cluster most similar to a vegetation spectrum is selected and the segmentation mask is shrunken to avoid extraction of mixed pixels at object border, furthermore specular highlights are detected and removed from the segmentation mask.


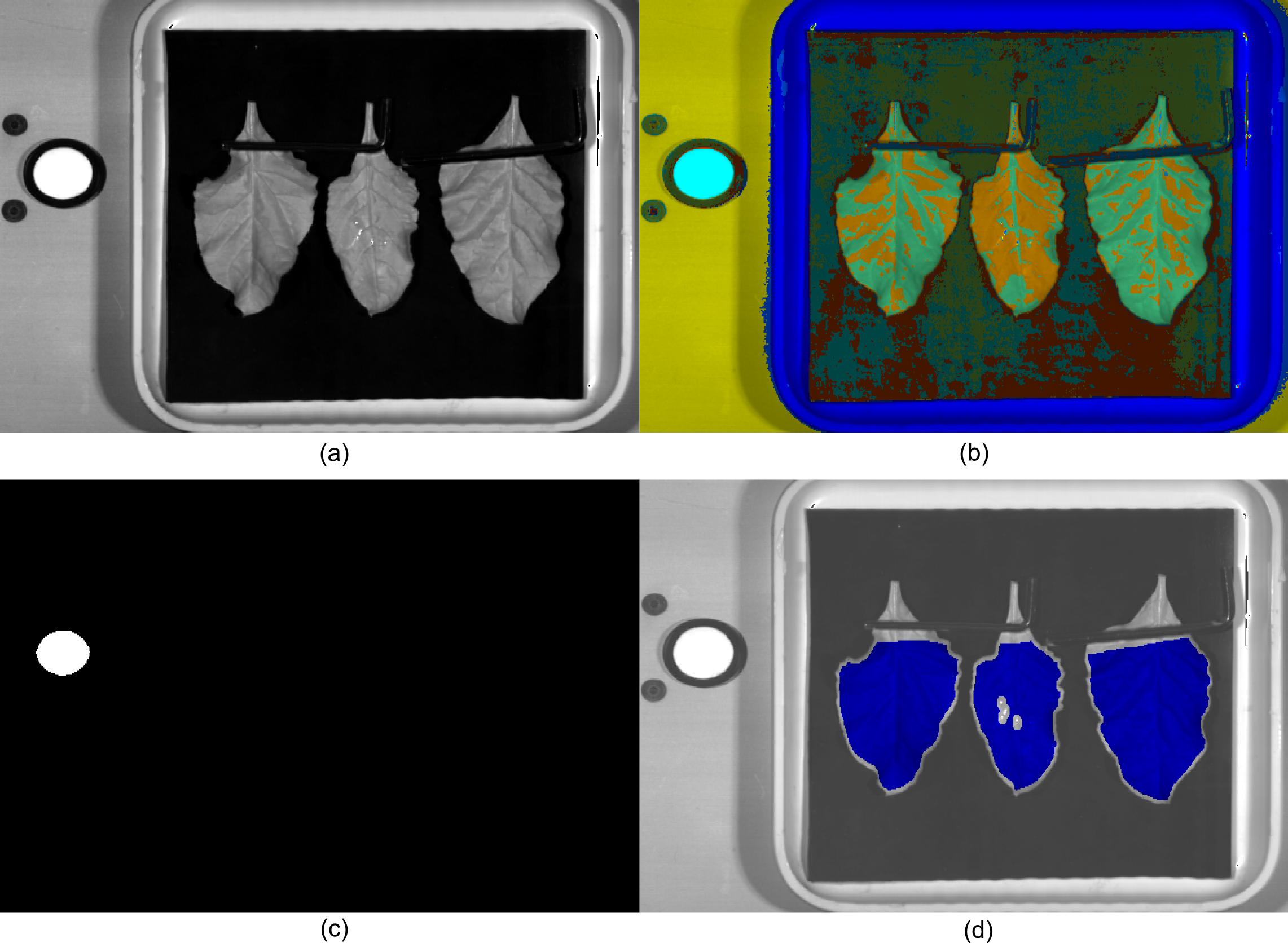


**Supplementary Figure 3. Correlation of the amount of fungal DNA determined by absolute and relative quantification**

Absolute quantification was based on a standard curve of fungal DNA. Relative quantification includes the amount of plant DNA by calculating a ∆ct. The coefficient of determination (R²) for the correlation of both methods was 0.97.

**Supplementary Figure 4.** Mass spectrum of peak A

**
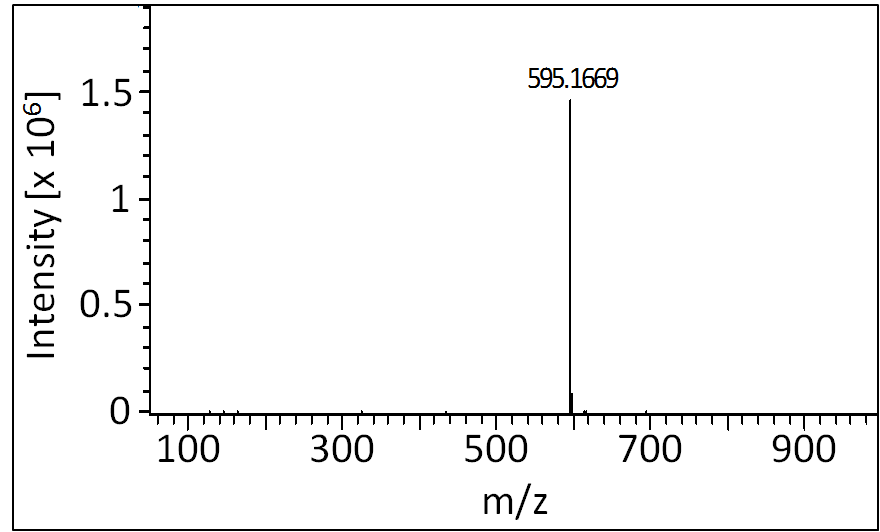
**

**Supplementary Figure 5.** The distribution of cultivar-specific differentially abundant features.

**Supplementary Table 1** The 58 differentially abundant features identified in the contrast between non-infected and infected plants, for which the LC-MS outcome was well correlated (R2 ≥0.7) with the hyperspectral data. (S) Compounds confirmed with reference standards; color code for cultivars (resistant: blue, susceptible: purple, tolerant: pink)

| # | RT(min): observed mass (m/z) | P< 0.05 (FWER) | Fold change | Molecular formula (M) | Adduct | Metabolite name | MS/MS fragments (m/z) | Metfusion score | In source | ^15^N Labeling | Hyper-  spectral R^2^ |
| --- | --- | --- | --- | --- | --- | --- | --- | --- | --- | --- | --- |
| 1 | 0.61 : 567.193 | 3.3E-03 | -1.52 | C23H34O16 | n/a | n/a | n/a | n/a | n/a | n.d. | 0.80 |
| 2 | 0.62 : 298.129 | 2.5E-03 | -2.90 | C14H19NO6 | M+Na | n/a | n/a | n/a | of 316 | n.d. | 0.73 |
| 4 | 0.63 : 198.076 | 3.8E-04 | -3.60 | C9H11NO4 | M+H | L-dopa^S^ | eV 35 (107.05; 123.04; 135.04; 152.07) | n/a | n/a | N1 | 0.80 |
| 5 | 0.67 : 175.024 | 2.8E-07 | 2.96 | C6H6O6 | M | n/a | n/a | n/a | of 193 | N0 | 0.73 |
| 6 | 0.67 : 193.035 | 5.5E-06 | 2.94 | C6H8O7 | M+H | Isocitric acid^S^ | n/a | n/a | n/a | N0 | 0.71 |
| 7 | 0.76 : 198.076 | 1.4E-03 | -2.99 | C9H12NO4 | M+H | dopa^D^ | eV 35 (107.05; 123.04; 135.04; 152.07) | n/a | n/a | N1 | 0.94 |
| 8 | 0.76 : 346.113 | 1.6E-08 | 2.01 | C14H19NO9 | M+H | n/a | eV 35 (154.09; 137.06; 121.07; 91.05) | n/a | n/a | N1 | 0.85 |
| 9 | 0.76 : 346.114 | 3.1E-05 | 2.52 | C14H19NO9 | M+H | n/a | eV 35 (154.09; 137.06; 121.07; 91.05) | n/a | n/a | N1 | 0.80 |
| 10 | 0.76 : 591.052 | 2.0E-08 | 2.10 | n/a | n/a | n/a | n/a | n/a | n/a | n.d. | 0.77 |
| 11 | 0.77 : 129.018 | 8.5E-05 | 3.11 | C5H4O4 | n/a | n/a | n/a | n/a | of 193; 215 | N0 | 0.75 |
| 12 | 0.77 : 157.013 | 9.9E-04 | 2.48 | C6H4O5 | n/a | n/a | n/a | n/a | of 193; 215 | N0 | 0.72 |
| 13 | 0.77 : 175.024 | 6.4E-04 | 4.49 | C6H6O6 | M+H | n/a | n/a | n/a | of 193; 215 | N0 | 0.88 |
| 14 | 0.77 : 215.016 | 4.6E-03 | 3.45 | C6H8O7 | M+Na | Citric acid^S^ | n/a | n/a | n/a | N0 | 0.74 |
| 15 | 0.77 : 316.140 | 9.3E-05 | -1.69 | C14H21NO7 | n/a | n/a | eV 20 (298.13; 154.09; 140.07; 137.06; 134.05; 121.07 119.05) | n/a | n/a | N1 | 0.77 |
| 16 | 0.77 : 591.053 | 1.2E-07 | 2.99 | n/a | n/a | n/a | n/a | n/a | n/a | n/a | 0.86 |
| 17 | 0.78 : 175.023 | 4.8E-03 | 4.36 | C6H6O6 | M+H | n/a | n/a | n/a | of 193; 215 | N0 | 0.79 |
| 18 | 0.78 : 215.016 | 3.7E-05 | 3.25 | C6H8O7 | M+Na | Citric acid^S^ | n/a | n/a | n/a | N0 | 0.87 |
| 19 | 0.78 : 393.983 | 1.4E-05 | 3.95 | n/a | n/a | n/a | n/a | n/a | n/a | N0 | 0.76 |
| 20 | 0.90 : 163.075 | 3.9E-04 | -1.77 | C10H10O2 | M+H | n/a | n/a | n/a | n/a | n/a | 0.87 |
| 21 | 1.32 : 220.118 | 4.3E-06 | -1.78 | C9H18NO5 | M+H | Pantothenic acid (Vitamin B5) ^S^ | eV 10 (202.11; 184.1) | 1.313 | n/a | N1 | 0.91 |
| 22 | 1.32 : 220.118 | 8.8E-05 | -2.05 | C9H17NO5 | M+H | Pantothenic acid (Vitamin B5) ^S^ | eV 10 (202.11; 184.1) | 1.313 | n/a | N1 | 0.88 |
| 23 | 3.14 : 145.029 | 1.3E-06 | -1.79 | C9H4O2 | M+H | n/a | n/a | n/a | n/a | n/a | 0.80 |
| 24 | 3.14 : 177.055 | 8.0E-05 | -1.76 | C10H8O3 | M+H | 1,3,8-Naphthalenertriol | eV 35 (89.039; 134.036; | 0.696 | n/a | N0 | 0.76 |
| 25 | 3.51 : 163.075 | 1.5E-05 | 1.55 | C10H10O2 | M+H | n/a | eV 15 (103.05; 131.05) | 1.05 | n/a | N0 | 0.89 |
| 26 | 3.65 : 177.055 | 4.4E-03 | -1.67 | C10H8O3 | M+H | n/a | eV 35 (89.04; 134.04; 117.04; 105.03) | 0.629 | n/a | N0 | 0.76 |
| 27 | 3.67 : 177.055 | 3.5E-03 | -1.63 | C10H8O3 | M+H | n/a | eV 35 (89.04; 134.04; 117.04; 105.03) | 0.629 | n/a | N0 | 0.80 |
| 28 | 3.94 : 161.060 | 6.6E-05 | 2.17 | C10H8O2 | M+H | n/a | n/a | n/a | n/a | N0 | 0.76 |
| 29 | 3.94 : 193.086 | 4.2E-06 | 2.10 | C11H12O3 | M+H | n/a | n/a | n/a | n/a | N0 | 0.88 |
| 30 | 3.94 : 372.141 | 2.5E-07 | 2.09 | C15N21N3O8 | M+H | n/a | n/a | n/a | n/a | n/a | 0.85 |
| 31 | 4.01 : 442.092 | 2.3E-03 | 1.51 | n/a | n/a | n/a | n/a | n/a | n/a | N0 | 0.87 |
| 32 | 4.02 : 149.096 | 8.3E-05 | 1.99 | C10H12O | M | n/a | n/a | n/a | of 411, 227 | N0 | 0.82 |
| 33 | 4.02 : 227.128 | 1.2E-04 | 2.24 | C12H18O4 | M | n/a | eV 10 (209.12; 191.11; 167.11; 149.10;131.09) | n/a | of 411 | N0 | 0.76 |
| 34 | 4.03 : 149.096 | 6.8E-05 | 1.57 | C10H12O | M | n/a | n/a | n/a | of 411, 227 | N0 | 0.84 |
| 35 | 4.03 : 163.112 | 1.1E-03 | 1.53 | C11H14O | n/a | n/a | n/a | n/a | of 411, 227 | n/a | 0.89 |
| 36 | 4.03 : 167.107 | 2.2E-03 | 1.68 | C10H14O2 | M | n/a | n/a | n/a | of 411, 227 | N0 | 0.91 |
| 37 | 4.03 : 191.107 | 6.8E-05 | 1.63 | C12H14O2 | M | n/a | n/a | n/a | of 411, 227 | N0 | 0.83 |
| 38 | 4.03 : 191.107 | 1.0E-05 | 1.94 | C12H14O2 | M | n/a | n/a | n/a | of 411, 227 | N0 | 0.74 |
| 39 | 4.03 : 227.128 | 1.7E-04 | 1.85 | C12H18O4 | M+H | n/a | eV 10 (209.12; 191.11; 167.11; 149.10;131.09) | n/a | of 411 | N0 | 0.80 |
| 40 | 4.03 : 411.162 | 1.5E-05 | 1.68 | C18H28O9 | M+Na | 12-hydroxyjasmonic acid 12-O-beta-D-glucoside | eV 10( 227.13 (249 M+Na) | 1 (Metfrag) | n/a | N0 | 0.90 |
| 41 | 4.03 : 411.162 | 9.0E-04 | 1.97 | C18H28O9 | M+Na | 12-hydroxyjasmonic acid 12-O-beta-D-glucoside; | eV 10( 227.13 (249 M+Na) | 1 (Metfrag) | n/a | N0 | 0.79 |
| 42 | 4.03 : 442.092 | 3.4E-03 | 1.51 | n/a | n/a | n/a | n/a | n/a | n/a | N0 | 0.87 |
| 43 | 4.03 : 442.092 | 7.0E-04 | 1.93 | C25H12O7 | n/a | n/a | n/a | n/a | n/a | N0 | 0.82 |
| 44 | 4.04 : 131.086 | 4.1E-03 | 1.52 | C10H10 | M | n/a | n/a | n/a | of 411, 227 | N0 | 0.81 |
| 45 | 4.05 : 131.086 | 2.2E-04 | 1.70 | C10H10 | M | n/a | n/a | n/a | of 411, 227 | N0 | 0.83 |
| 46 | 4.05 : 209.117 | 3.1E-03 | 1.56 | C12H16O3 | M | n/a | n/a | n/a | of 411, 227 | N0 | 0.93 |
| 47 | 4.11 : 227.128 | 3.2E-03 | 1.57 | C12H18O4 | M+H | n/a | eV 10 (209.12; 191.11; 167.11; 149.10;131.09) | n/a | of 411 | N0 | 0.71 |
| 48 | 4.20 : 122.027 | 2.8E-05 | 1.70 | C3H7NO2S | n/a | n/a | n/a | n/a | n/a | N1 | 0.79 |
| 49 | 4.20 : 295.111 | 5.0E-06 | 1.64 | n/a | n/a | n/a | n/a | n/a | n/a | n/a | 0.81 |
| 50 | 4.20 : 416.131 | 3.9E-05 | 1.97 | n/a | n/a | n/a | n/a | n/a | n/a | n/a | 0.87 |
| 51 | 6.40 : 135.117 | 1.0E-03 | 1.53 | C10H14 | n/a | n/a | n/a | n/a | n/a | N0 | 0.76 |
| 52 | 6.77 : 131.086 | 2.6E-04 | 2.18 | C10H10 | n/a | n/a | n/a | n/a | n/a | n/a | 0.78 |
| 53 | 6.77 : 276.196 | 1.4E-05 | 2.23 | C17H25NO2 | n/a | n/a | n/a | n/a | n/a | N1 | 0.80 |
| 54 | 6.77 : 304.191 | 4.3E-05 | 2.56 | C18H25NO3 | n/a | n/a | n/a | n/a | n/a | N1 | 0.88 |
| 55 | 6.77 : 362.194 | 4.3E-04 | 2.26 | C18H29NO5 | M+Na | n/a | n/a | n/a | n/a | N1 | 0.77 |
| 56 | 6.78 : 132.102 | 8.6E-04 | 2.23 | C6H13NO2 | n/a | n/a | n/a | n/a | n/a | N1 | 0.75 |
| 57 | 7.49 : 177.055 | 2.5E-04 | -2.10 | C10H8O3 | M+H | n/a | eV 35 (134.0385; 89.0386) | 0.71 | n/a | N0 | 0.71 |
| 58 | 7.58 : 369.119 | 3.1E-03 | 3.34 | C17H20O9 | M+H | 5-O-Feruloylquinic acid | eV 35 (207.07; 175.04; 147.04; 119.05; 91.06 | 0.83 | n/a | N0 | 0.76 |
